# Non-fumigant Nematicides are Promising Alternatives to Fumigants for the Management of *Meloidogyne Enterolobii* in Tobacco

**DOI:** 10.2478/jofnem-2022-0045

**Published:** 2022-11-09

**Authors:** M. S. Alam, C. Khanal, W. Rutter, J. Roberts

**Affiliations:** 1 Department of Plant and Environmental Sciences, Clemson University, Clemson, SC 29643 Carolina; 2 USDA-ARS, U.S. Vegetable Laboratory, Charleston, SC 29643 Carolina

**Keywords:** biological, *Burkholderia*, fluensulfone, fluopyram, management, *Meloidogyne enterolobii*, nematicide, non-fumigant, oxamyl, tobacco

## Abstract

Experiments were conducted to evaluate the efficacy of three currently available non-fumigant chemical nematicides (oxamyl, fluopyram, and fluensulfone) and a biological nematicide derived from *Burkholderia* against *Meloidogyne enterolobii* on tobacco in a growth room environment. The non-fumigant chemical nematicides greatly suppressed nematode egg production compared to the untreated control, the suppression being 99.9% for fluensulfone and oxamyl, and 93% for fluopyram. Similarly, oxamyl-, fluensulfone-, and fluopyram-treated pots, respectively, had 99%, 98%, and 94% less J2/100 cm^3^ of soil than those in the control. The biological nematicide did not have a significant effect on nematode egg production and the soil abundance of J2. The root biomass of tobacco was significantly reduced by the application of fluensulfone, while the effects of oxamyl, fluopyram, and *Burkholderia* metabolites were not significant compared to the untreated control. Results from this study suggest that non-fumigant nematicides have a potential to serve as an alternative to fumigant nematicides.

The guava root-knot nematode (*Meloidogyne enterolobii* Yang and Einenback, 1983) is a cosmopolitan, polyphagous, and highly damaging species of root-knot nematode. Tobacco (*Nicotiana tabacum* L.) is a known host of *M. enterolobii*, although the extent of damage (qualitative and quantitative) the nematode can cause to this crop has not been studied well. Being a specialty crop with field production in restricted geography and limited funding opportunities for research could be the reasons behind the scarcity of tobacco– nematode interaction studies. Nevertheless, reports of *M. enterolobii* infestations in tobacco fields in North Carolina (Ye, 2018) and Brazil (Filho *et al*., 2016) suggest the need for studies on the reproduction, pathogenicity, and management of the nematode in order to save the 15-billion-dollar tobacco industry (FAO, 2019).

As seen with many other agricultural crops, *M. enterolobii* management in tobacco is challenging. A very wide host range and ability to overcome resistance effective for other common species of root-knot nematodes make *M. enterolobii* unmanageable with currently available nematode management practices (Brito *et al*., 2007; Kiewnick *et al*., 2009; Rutter *et al*., 2019; Ye *et al*., 2021; Khanal and Harshman, 2022). The use of fumigant nematicides is the most common method of nematode management in commercial agricultural production systems; however, none of the currently available fumigants are able to provide satisfactory management of *M. enterolobii*. Additionally, with the increasing cost of fumigants, their inability to protect crops from nematodes throughout the crop growing season, increased legal restrictions, and adverse effects on human health and the environment render their use less preferred, cumbersome, and unsustainable (Khanal *et al*., 2018, 2021; Khanal and Desaeger, 2020). The undesirable consequences of fumigant nematicides have led to the development of safer non-fumigant nematicides by chemical industries over the past couple of decades. Additionally, some biological nematicides have also been released. While the use of non-fumigant nematicides can be an alternative to fumigants, published studies on their efficacy against *M. enterolobii* in tobacco are not available. Additionally, the efficacy of non-fumigant nematicides can greatly vary depending on the nematode species, host crop, and season (Oka and Saroya, 2019; Khanal and Desaeger, 2020; Watson, 2022). The objective of the current study was to evaluate the efficacy of three non-fumigant chemical nematicides (oxamyl, Vydate^®^ L, Corteva Agriscience, Indianapolis, IN; fluopyram, Velum^®^ Prime, Bayer CropScience, Research Triangle Park, NC; and fluensulfone, Nimitz^®^, ADAMA Agricultural Solutions Ltd., Raleigh, NC) and one biological nematicide derived from *Burkholderia* metabolites (Majestene^®^, Marrone Bio Innovations, Davis, CA) on reproduction and pathogenicity of *M. enterolobii* in tobacco.

## Materials and Methods

### Preparation of nematode inoculum

Pure cultures of *M. enterolobii* were maintained in a growth room environment on tomato (*Solanum lycopersicum* L., cv. Rutgers, Seedway, Hall, NY). Nematode eggs were extracted by agitating tomato roots in 0.6% NaOCl for 4 min, as described by Hussey and Barker (1973). The extracted eggs were transferred to a modified Baermann pan in an incubator (VWR, Cornelius, OR) at 25°C. Second-stage juveniles (J2) collected 2 d after incubation served as inoculum for this study.

### Establishment of experiments

Experiments were conducted in a growth room because of the quarantine nature of the pathogen that requires a Biosafety Level-2 facility. Experiments were established in 15-cm-top diameter plastic pots containing 1.5 kg sandy loam soil steam sterilized for 5 hr at 135°C prior to use. The experiment was established as a randomized block design with five replications. Treatments included a single application of the currently recommended rate of three non-fumigant chemical nematicides (oxamyl, fluopyram, and fluensulfone) and a biological nematicide (*Burkholderia* metabolites) plus an untreated control (Table 1). Each pot received an 8-wk-old tobacco seedling (cultivar K 346, Gold Leaf Seed Company, Hartsville, SC) with four to six true leaves. Each plant was inoculated with an aqueous suspension of 1,000 freshly hatched J2 of *M. enterolobii* on the day of transplanting. The inoculum was pipetted into three 0.5-cm-diam. × 5-cm deep depressions arranged into a triangular pattern and 2-cm away from the crown region. Nematicides were drench applied 2 d after inoculation, except for the pots receiving fluensulfone, which were planted a week after nematicide application because of possible phytotoxicity. Standard fertilization and insect management practices were conducted. The average daily room temperature during the study period was 30 ± 5°C. The relative humidity of the growth room was 44 ± 4% during the study period. Four metal halide bulbs (1,000 W) hanging approximately 3 m above the table provided a 12-hr photoperiod.

**Table 1:** Non-fumigant chemical and biological nematicide treatments and their recommended application rates used in the current study.

Treatment	Application rate (L/ha)
Oxamyl	4.68
Fluopyram	0.50
Fluensulfone	4.09
Burkholderia^a^	18.71
Untreated control	–

a *Burkholderia rinojensis* strain A396 secondary metabolites.

The experiments were terminated 60 d after inoculation. Eggs were extracted from whole root systems in each pot on the day of termination by agitating roots in 0.6% NaOCl for 4 min to dislodge eggs from egg masses (Hussey and Barker, 1973). The eggs were enumerated within 24 hr of extraction using a compound microscope (Martin Microscope Company, Easley, SC) at 40× magnification. The soil samples were placed in a walk-in cooler at 4°C until nematode extraction. Nematode J2 were extracted from 100 cm^3^ subsample of soil from each pot using the centrifugal flotation technique (Jenkins, 1964). Extraction and enumeration of J2 were conducted within 2 wk of termination of the experiments. The J2 and eggs were enumerated using a compound microscope (Martin Microscope Company) at 40× magnification. The aboveground parts (shoot) and belowground parts (root) were dried at 45°C for 2 wk, and dry biomass was recorded for the determination of pathogenicity of the nematode on tobacco. The entire experiment was repeated once.

### Data analysis

Data were subject to one-way analysis of variance using the mixed model in JMP PRO 16.0 (SAS Institute, Cary, NC). Nematode reproduction data were subjected to log transformation (log x + 1) before analysis. Nematicide treatment was used as a fixed effect, and replication was used as a random effect. Tukey’s HSD or Student’s *t*-test (*P* ≤ 0.05) was used as *post-hoc* mean comparisons.

## Results

**Figure 1:**
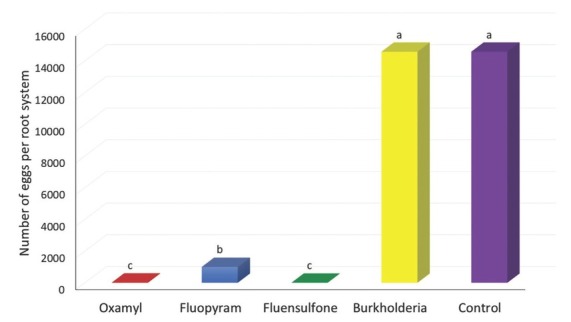
Reproduction of *Meloidogyne enterolobii* expressed as number of eggs per root system of tobacco inoculated with 1,000 freshly hatched second-stage juveniles, treated with non-fumigant nematicides, and harvested at 60 d after inoculation. Data were combined over two experiments and are means of 10 replications. Within columns, means followed by a common letter are not significantly different according to Tukey’s HSD test (*P* ≤ 0.05). Burkholderia refers to *Burkholderia rinojensis* strain A396 secondary metabolites.

**Figure 2:**
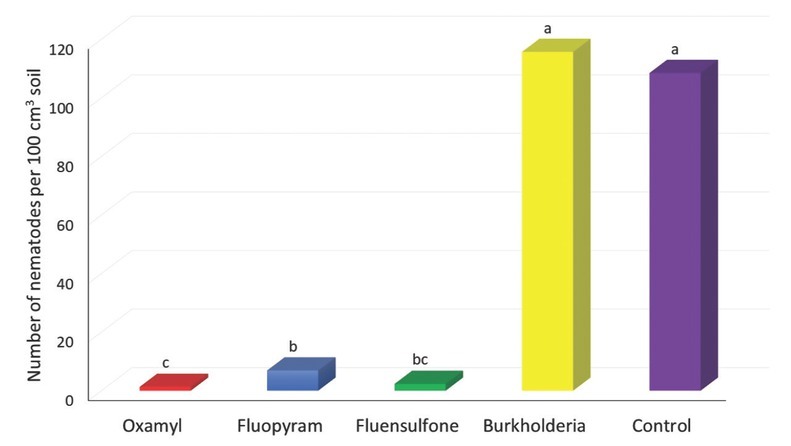
Reproduction of *Meloidogyne enterolobii* expressed as number of second-stage juveniles per 100 cm^3^ of soil containing tobacco inoculated with 1,000 freshly hatched second-stage juveniles, treated with non-fumigant nematicides, and harvested at 60 d after inoculation. Data were combined over two experiments and are means of 10 replications. Within columns, means followed by a common letter are not significantly different according to Student’s *t*-test (*P* ≤ 0.05). Burkholderia refers to *Burkholderia rinojensis* strain A396 secondary metabolites.

**Figure 3:**
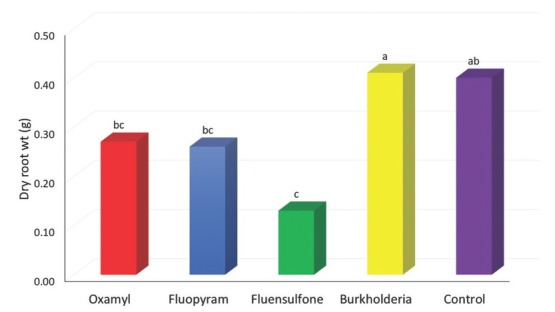
Dry weights of tobacco roots 60 d after inoculation with 1,000 freshly hatched second-stage juveniles of *Meloidogyne enterolobii* and application of non-fumigant nematicides. Data are means of 10 replications. The plant material was dried at 45°C for 2 wk. Bars with common letters are not significantly different according to Tukey’s HSD test (*P* ≤ 0.05).

Data from two experiments were combined for analysis because of the absence of significant treatment by experiment interactions. The nematicides had a significant effect on nematode reproduction (J2 and eggs) and plant root weight (Figs. 1–3), but not on plant shoot weight (*P* = 0.5006).

The effect of nematicides on nematode reproduction expressed as number of eggs per root system is presented in Figure 1. The nematode reproduction ranged from 6 to 14,576 eggs per root system. All non-fumigant chemical nematicides (oxamyl, fluopyram, and fluensulfone) significantly suppressed the egg production compared to the control, with fluensulfone having the greatest suppression (99.96%), followed by oxamyl (99.91%) and fluopyram (93%). The number of nematode eggs in pots treated with the *Burkholderia* metabolites was statistically similar to that in the control.

The effect of nematicides on nematode reproduction expressed as number of J2 per 100 cm^3^ soil was similar to the effect on the number of eggs, as presented in Figure 2. The nematode reproduction ranged from 1 to 116 J2/100 cm^3^ of soil. Nematode reproduction was significantly suppressed by oxamyl, fluopyram, and fluensulfone compared to that in control, the suppression being 99% for oxamyl, 98% for fluensulfone, and 94% for fluopyram. *Burkholderia* metabolites did not have a significant effect on the nematode reproduction compared to the control.

Tobacco root biomass was significantly affected by the application of nematicides, as presented in Figure 3. Fluensulfone significantly reduced the root dry weight compared to the control, the reduction being 68%. Although not significantly different from the control, tobacco treated with oxamyl and fluopyram had 33% and 35% lower root dry weight, respectively. The root dry weight of tobacco treated with *Burkholderia* metabolites did not differ significantly from that of control.

## Discussion

*Meloidogyne enterolobii* has drawn great attention from nematologists in recent years due to its large host range, aggressiveness, and management challenge. Until recently, 95% of crops lost to root-knot nematodes would be attributed to four species of root-knot nematodes, namely, *M. incognita*, *M. arenaria*, *M. javanica*, and *M. hapla*. However, crop lost to *M. enterolobii* will likely share the greatest portion of total crop losses attributed to root-knot nematodes because of its rapid geographic expansion and the lack of effective management methods (Khanal and Harshman, 2022). Additionally, a high reproduction rate and pathogenicity of *M. enterolobii* may outcompete other root-knot nematode species present in the field, making its management a further challenge.

Most studies on the management of *M. enterolobii* are focused on row and vegetable crops. Being a minor crop, tobacco has received little to no attention for management against *M. enterolobii*; however, due to its economic importance, the effect of this nematode on tobacco should be further studied. It is a common understanding of the growers that application of fumigants helps manage soil-borne pathogens including nematodes; however, none of the currently available fumigant nematicides are effective in managing *M. enterolobii*. Additionally, adverse effects of fumigant nematicides on human health and the environment, increasing cost due to the legal requirement of the fumigant management plan render fumigant nematicides less desirable (Khanal and Desaeger, 2020). Of the four non-fumigant nematicides evaluated in the present study, three chemical nematicides significantly suppressed *M. enterolobii*, suggesting these nematicides can be a potential alternative to fumigant nematicides. Oxamyl and fluensulfone were the most effective nematicides in suppressing nematode eggs, followed by fluopyram. Similarly, the three chemical non-fumigants greatly suppressed the soil abundance of nematode relative to the control. A similar effect of non-fumigant nematicides was observed in a growth cabinet experiment conducted by Watson (2022) who reported that fluopyram, fluensulfone, and oxamyl significantly suppressed *M. enterolobii* egg production. While the chemical non-fumigants were very effective in suppressing nematode reproduction, the current study did not find any effects of the *Burkholderia* metabolites on nematode reproduction. Although 93% to 99.9% nematode reproduction was suppressed by the non-fumigant chemical nematicides compared to the control, this study suggests that the currently recommended rate of these nematicides is not enough to eradicate *M. enterolobii*. More studies are needed to determine higher efficacy rates.

Reproduction of a nematode in a host crop is an indicator of host susceptibility to the nematode. The reproduction of *M. enterolobii* on the untreated control was 14,576 eggs per root system, which is approximately 13 times lower than that reported by Khanal and Harshman (2022) on tomato, suggesting the possibility of presence of some *M. enterolobii*suppressive mechanisms in tobacco. A lower reproduction rate of the nematode on tobacco was also reported by Adamo *et al*. (2021) who found the highest amount of 11,612 eggs per fresh gram of tobacco root, although that study employed a different nematode species (*M. arenaria*) and different tobacco cultivars. The tobacco cultivar K 346 is resistant to *M. incognita* races 1 and 3 and many other diseases; however, any resistance to *M. enterolobii* is not known. Studies are needed to determine the nematode suppression mechanism in tobacco.

An ideal nematicide, although not available, should keep the nematode population below the damage threshold while exhibiting no adverse effects on the host plant. The current study showed that the non-fumigant chemical nematicides not only greatly suppressed *M. enterolobii* reproduction but also reduced the tobacco root biomass numerically or significantly, suggesting a trade-off between nematode suppression and host plant biomass likely exists. However, the shoot weight of tobacco was not significantly reduced by the application of the chemical nematicides, implying these nematicides can be a good choice for the growers as tobacco is grown for the shoots. Although oxamyl, fluopyram, and fluensulfone have been reported to have low to high systemic activity in plants (Alphey *et al*., 1975; Oka *et al*., 2012; Jeschke, 2017), results from the current study suggest they are likely concentrated more in the root system. However, studies are needed to confirm the translocation and variable concentrations of nematicides in different plant parts. Furthermore, while the current study measured quantitative losses, further studies are needed to determine if any qualitative losses are possible from the application of the non-fumigant nematicides on tobacco.
